# LncRNA UCA1 Promotes the Progression of AML by Upregulating the Expression of CXCR4 and CYP1B1 by Affecting the Stability of METTL14

**DOI:** 10.1155/2022/2756986

**Published:** 2022-02-08

**Authors:** Jiajia Li, Zhongyu Li, Xue Bai, Xiaofeng Chen, Meng Wang, Yanping Wu, Haotian Wu

**Affiliations:** ^1^Department of Hematology, The First Affiliated Hospital of Bengbu Medical College, Bengbu, Anhui 233000, China; ^2^Bengbu Medical College, Bengbu, Anhui 233030, China

## Abstract

**Objective:**

Increasing numbers of studies have proved that m6A methylation plays crucial roles in different cancers. However, how lncRNA regulates m6A methylation and participates in acute myeloid leukemia (AML) remains unclear. Therefore, this study aims to explore the function and mechanism of UCA1 in AML by regulating m6A methylation.

**Methods:**

qRT-PCR, western blot, and immunohistochemical staining were used to detect the expression of METTL14, CXCR4, and CYP1B1. qRT-PCR was used to detect the expression of UCA1. CCK8, flow cytometry, and transwell assays were used to detect the proliferation, apoptosis, migration, and invasion of HL60 and U937 cells, respectively. m6A methylation was detected by dot blot analysis. Tumor-bearing mice were established, and tumor weight and volume were analyzed. Immunofluorescence staining, co-localization, and RNA pull-down were used to confirm the reaction between UCA1 and METTL14.

**Results:**

Overexpression of UCA1 promotes AML development *in vitro*. Furthermore, we found that METTL14-influenced m6A methylation could be affected by UCA1. UCA1 promoted AML development by regulating m6A methylation. Moreover, the expression of CYP1B1 and CXCR4 was affected by METTL14. In addition, UCA1 promoted AML development by affecting m6A methylation *in vivo*.

**Conclusion:**

In the present study, we demonstrated that lncRNAUCA1 promotes the progression of AML by upregulating the expression of CXCR4 and CYP1B1 by affecting the stability of METTL14.

## 1. Introduction

Acute myeloid leukemia (AML) is a malignant tumor of the blood system and is the most common type of adult acute leukemia [1]. Chemotherapy is currently the main treatment for AML, which has lasted for nearly 40 years, with a low cure rate and easy relapse [1]. Therefore, AML is a highly heterogeneous myeloid tumor with malignant clonal hyperplasia of HSPCs. It is characterized by enhanced self-renewal, uncontrolled proliferation, hindered differentiation, and suppressed apoptosis [2]. Therefore, it is urgent to explore the pathogenesis of AML and to develop new treatment strategies in order to reduce chemotherapy-related side effects and improve the overall survival of patients.

The pathogenesis of AML is complicated due to the disorder of gene regulation, cell differentiation, proliferation, and apoptosis, which ultimately leads to the malignant transformation of hematopoietic stem and progenitor cells [3]. With the rapid development of next-generation gene-sequencing technology, more and more long non-coding RNA (lncRNA) have been found to be closely related to the occurrence and development of AML [4, 5]. Sun et al. demonstrated that lncRNA ANRIL regulates AML development through modulating the glucose metabolism pathway of AdipoR1/AMPK/SIRT1 [6]. Feng et al. revealed that lncRNA NR-104098 inhibits AML proliferation and induces differentiation through repressing EZH2 transcription by interacting with E2F1 [7]. Yang et al. demonstrated that overexpression of lncRNA PANDAR predicts adverse prognosis in acute myeloid leukemia [8]. In the previous study, we demonstrated that lncRNA UCA1 modulates cell proliferation and apoptosis by regulating miR-296-3p/Myc axis in acute myeloid leukemia [9]. However, the mechanism of UCA1 in AML development remains unclear.

N-6-methyladenosine (m6A) methylation is the most common RNA modification in mammals, which regulates the expression of genes after transcription without changing the base sequence. This modification process is reversible; is jointly participated by methyltransferases (writers), demethylases (erasers), and methylated readers (readers); and is widely involved in mammalian reproductive development, immunity, metabolism, and tumors [10]. It mainly participates in many biological processes and diseases through three types of proteins, including methyltransferase, methylation reading protein, and demethylase [11]. Emerging evidence suggests that m6A methylation plays a critical role in cancer through various mechanisms and has provided more possibilities for the early diagnosis and treatment of cancers [12, 13]. FTO is highly expressed in AMLs, enhances leukemic oncogene-mediated cell transformation and leukemogenesis, and inhibits all-trans retinoic-acid-induced AML cell differentiation, through regulating the expression of targets such as ASB2 and RARA by reducing m6A levels in these mRNA transcripts [14]. Chen et al. demonstrated that m6A on mRNA facilitated a phase-separated nuclear body that suppresses myeloid leukemia cell differentiation [15]. In the present study, we found that the expression of METTL14 was significantly downregulated when suppressing UCA1, suggesting that UCA1 promotes AML development by regulating METTL14 expression. Therefore, this study aims to explore the function and mechanism of UCA1 on AML development via regulating METTL14-guided m6A methylation.

## 2. Materials and Methods

### 2.1. Patients and Samples

A total of nine diagnosed patients with AML participants and nine healthy controls were recruited by the First Affiliated Hospital of Bengbu Medical College from 2019 to 2021. Peripheral venous blood samples were collected from each participant at the same time. All participants signed informed consent forms, and the research protocol was approved by the Ethics Committee of the First Affiliated Hospital of Bengbu Medical College.

### 2.2. Cell Culture, Transfection, and Treatment

HL60 and U937 cells were purchased from Hasenbio and cultured in Dulbecco's modified Eagle's medium (DMEM, Invitrogen) supplemented with 10% fetal bovine serum, 100 units/ml of penicillin, and 100 *μ*g/ml of streptomycin at 37°C in 5% CO_2_. Transfection of over-UCA1-pcDNA3.1, over-METTL14-pcDNA3.1 plasmid, SiRNA of UCA1, and SiRNA of METTL14 was performed when the confluence of HL60 and U937 reached 60–70% using Lipofectamine 2000 (Invitrogen, 11668–027) following the manufacturer's instructions. Cells were collected for further analysis at 48 h after transfection. The SiRNA sequence of METTL14 or lncRNA UCA1 were as follows: UCA1 (HL60): sense–5′-GCACCUUGUUAGCUACAUAAA-3′ and antisense–5′-UAUGUAGCUAACAAGGUGCCA-3′; UCA1(U937): sense: 5′ –CAAAGAUCUGCAAUCAGAACU-3′ and antisense–5′-UUCUGAUUGCAGAUCUUUGUG-3′; METTL14 (HL60): sense–5′-CCUUCUUUAUUGUAAUUAAAU-3′ and antisense–5′-UUAAUUACAAUAAAGAAGGUU-3′; and METTL14 (U937): sense–5′-GGAUGAGUUAAUAGCUAAAUC-3′ and antisense–5′-UUUAGCUAUUAACUCAUCCUU-3′.

### 2.3. Quantitative Real-Time Polymerase Chain Reaction (qRT-PCR) Analysis

Total RNA was extracted from peripheral venous blood, HL60, and U937 cells or tissues using TRIzol (BS259A, Biosharp) and then reversely transcribed into cDNA using PrimeScript RT Reagent kit (R223-01, Vazyme). The real-time PCR assay was conducted with SYBR Green Real-Time PCR Master Mix (Q711-02, Vazyme). Actin acted as the control. The primers for UCA1, METTL14, CXCR4, CYP1B1, and *β-*actin were listed in Supplement Table 1. The relative gene expression was analyzed by the 2^−ΔΔCt^ method.

### 2.4. Western Blot Analysis

Total proteins were extracted from cells or tissues using RIPA lysis buffer (BL504A, Biosharp). Protein concentrations were determined by BCA assay (Bl521A, Biosharp). Thirty microgram proteins were subjected to SDS-PAGE and transferred onto PVDF. Subsequently, the membranes were incubated overnight at 4°C with primary antibodies after blocking in PBS with 5% skim milk. Membranes were subsequently incubated with goat anti-rabbit peroxidase-conjugated secondary antibodies (BL003A, Biosharp) at 37°C for 1 h. The immunoreactive bands were detected using the ECL kit (WBKLS0100, Millipore). The primary antibodies were as follows: CXCR4 (ab181020, Abcam), CYPB1 (ab32649, Abcam), METTL14 (ab252562, Abcam), and actin (GB12001, Servicebio).

### 2.5. CCK8 Assay

Cell survival was assessed using the CCK8 (Dojindo, Japan) assay. Briefly, HL60 and U937 were cultivated at subconfluence before being washed twice with phosphate-buffered saline (PBS) and then resuspended in a culture medium with FBS. Subsequently, the cells were plated at 2 × 10^3^ cells/well in 96-well microliter plates in 100 *μ*L media. The cells were washed after culturing for 48 h. The absorbance of MTT was measured at 492 nm using a Molecular Devices SpectraMax i3.

### 2.6. Apoptosis Assay by Flow Cytometry

HL60 and U937 were treated with over-UCA1-pcDNA3.1, over-METTL14-pcDNA3.1 plasmid, SiRNA of UCA1, and SiRNA of METTL14 and were cultured in a six-well plate, respectively. And then cells were double-stained with propidium iodide (PI) and annexin V (HS-SJ069, Hasenbio). Briefly, transfected HL60 and U937 cells were collected and resuspended in 500 *μ*L 1 × binding buffer. Then, cells were stained with 10 *μ*L annexin V-FITC and 5 *μ*L PI (50 *μ*g/mL) for 15 mins under dark conditions at room temperature. Cell apoptotic assay was determined by using a FACSVerse flow cytometry (BD Biosciences).

### 2.7. Cell Invasion/Migration Assays

For cell migration analysis, 2 × 10^5^ HL-60 or U937 cells were plated in medium without serum in the top chamber of a transwell (Corning), while the medium containing 20% FBS was placed in the lower well. The cells were fixed in 4% formaldehyde, stained by crystal violet dye for 10 min, and photographed under a microscope after 24 h incubation. Cell invasion assays were performed using Boyden chambers with 8 µm filter inserts coated with 40 *µ*g Matrigel in 24-well plate dishes, and the other procedure was the same to cell migration assays.

### 2.8. Soft Agar Assay for Colony Formation

Cells of 1 × 10^4^ HL60 or U937 were plated in 0.4% agarose on top of a 1% agarose base supplemented with a complete medium containing Phen (0.1 mM) or Met (10 mM) or in the blank medium. Cells in agarose were allowed to grow for 4 weeks in 5% CO_2_ at 37°C, and total colonies were counted. Pictures were taken, and the number of colonies was counted by a microscope.

### 2.9. Dot Blot Analysis

RNA samples were denatured and spotted to a nitrocellulose membrane under a vacuum. The membranes were blocked for 1 h in 5% nonfat dry milk in 0.1% PBST (HS-SJ021, Hasenbio) after UV cross-linking. Rabbit anti-m6A antibody was diluted 1:500 in 0.1% PBST and incubated with the membranes overnight (4°C). The blot was incubated with Dylight 800 AffiniPure goat anti-rabbit IgG (*H* + *L*) for 1 h at 25°C after washing with 0.1% PBST. The membranes were washed again with 0.1% PBST and scanned with an Odyssey infrared imaging system (Roche LightCycler^®^ 480II).

### 2.10. Tumor Xenograft in Nude Mice

Mice aged 4–6 weeks were purchased at Comparative Medicine Center of Yangzhou University and were fed in a specific pathogen-free grade animal laboratory for 1 week. Mice were randomly distributed into four groups, with five mice in each group. HL-60 or U937 cells with different treatments were prepared into 0.1 mL cell suspension (1 × 10^6^) and subcutaneously injected into the neck and back. The growth of the tumor was observed after 0, 7, 14, 21, and 28 days.

### 2.11. Immunofluorescent Staining

Cells were fixed by 4% paraformaldehyde for 15 minutes and then immersed 3 times in PBS. Subsequently, cells were permeated by 0.5% Triton X-100 at room temperature for 20 minutes. Immunofluorescent staining was conducted as follows. Cells were stained primary antibody against METTL14 and then cultured with the fluorescent secondary antibody (Cy3-conjugated donkey anti-goat antibody, 1:100, Santa Cruz). The slides were rinsed with PBS and mounted with Dako Cytomation. All images were visualized on a Leica SP5 confocal microscope (Leica TCS SP5) and were analyzed with Leica LAS AF software (Leica Wetzlar). For co-localization of UCA1 and METTL14, signals for fluorescence in situ hybridization of UCA1 and the immunofluorescent staining of METTL14 were overlapped.

### 2.12. RNA Pull-Down

RNA pull-down assays were performed using an RNA pull-down kit (Thermo Fisher, 20164) according to the instruction protocols. In brief, 10^5^ cells were washed in ice-cold phosphate-buffered saline, lysed in 500 *μ*L co-IP buffer, and incubated with 3 *μ*g biotinylated DNA oligo probes against UCA1 for 2 h at room temperature. A total of 50 *μ*L washed Streptavidin C1 magnetic beads (Invitrogen) were added to each binding reaction and further incubated at room temperature for half an hour. The beads were washed briefly with RIPA lysis buffer 5 times. The bound proteins in the pull-down materials were analyzed by western blot.

### 2.13. Immunohistochemical Staining

For immunohistochemical staining, tumor tissue sections were deparaffinized in xylene and dehydrated with graded ethanol. After washing with distilled water, tissue peroxidase was blocked with 3.0% hydrogen peroxide in methanol for 15 min at room temperature. After washing, the slides were incubated with primary antibodies against METTL14 (ab181020, Abcam), CXCR4 (ab181020, Abcam), and CYP1B1 (ab32649, Abcam) overnight at 4°C. The slides were washed with PBS and then incubated with biotinylated anti-mouse IgG (ZSGB-BIO, PV-9001) for 10 min at room temperature. The slides were washed with PBS again and then incubated with horseradish peroxidase-conjugated streptavidin (Jackson, 016-030-084) for 5 min at room temperature. For all slides, the immune reaction was demonstrated with DAB. The sections were then counterstained with Meyer's hematoxylin, dehydrated, and imaged by a microscope.

### 2.14. Statistical Analysis

All statistical analyses in the present study were performed using GraphPad Prism 5.0, and the data were represented as mean ± standard deviation (SD). Student's *t*-test or one-way analysis of variance (ANOVA) followed by Tukey's multiple comparisons test was used to determine the statistical significance between two and more than two groups. *P* < 0.05 was considered to indicate statistical significance.

## 3. Results

### 3.1. UCA1 Promoted AML Development *in Vitro*

**Figure 1:**
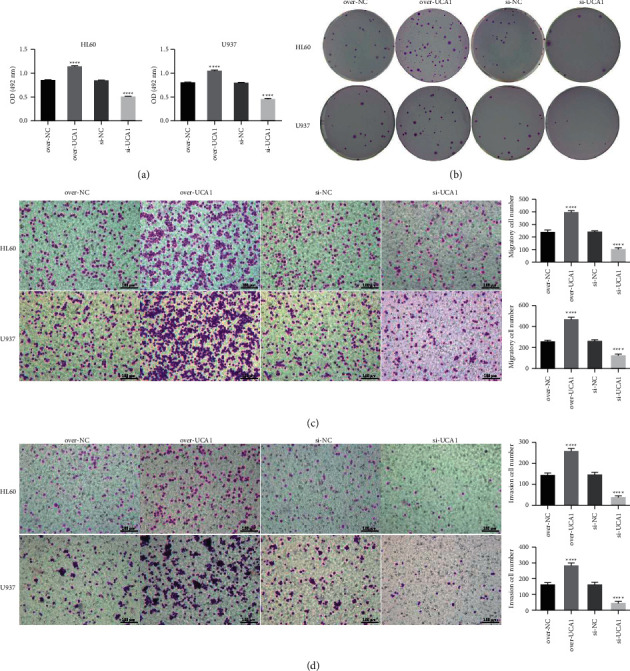
UCA1 promotes AML cell proliferation, migration, and invasion *in vitro*: (a) CCK8 assay was used to detect the HL60 and U973 cell proliferation affected by lncRNA; (b) colony formation was detected by colony formation assay affected by lncRNA UCA1 in HL60 and U973 cells; (c, d) transwell analysis was used to detect the invasion and migration of HL60 and U973 cells affected by lncRNA UCA1, respectively. The data were presented by mean ± SD. ^*∗∗∗∗*^*P* < 0.001.

**Figure 2:**
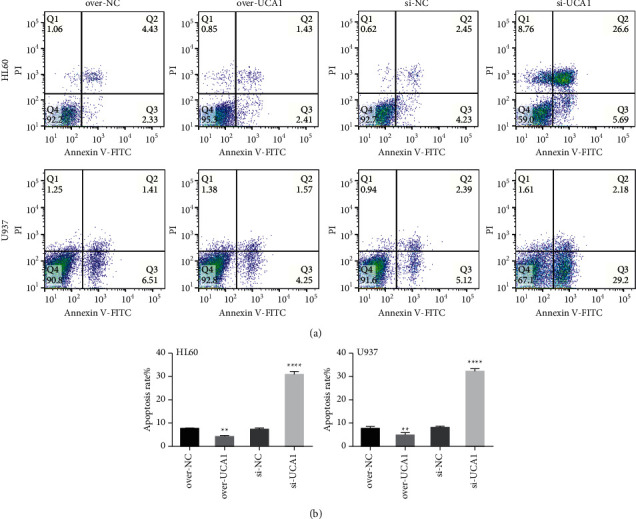
UCA1 suppressed AML cell apoptosis: (a) flow cytometry pictures showing the apoptosis of HL60 and U973 cell affected by lncRNA UCA1; (b) the quantitative data for cell apoptosis rate. The data were presented by mean ± SD. ^*∗∗*^*P* < 0.01 and ^*∗∗∗∗*^*P* < 0.001.

In the previous study, we have demonstrated that UCA1 has been identified as an oncogene and is involved in AML [16]. In the present study, the CCK8 assay showed that overexpression of UCA1 could promote HL60 and U973 cell proliferation, while suppression of UCA1 inhibited HL60 and U973 cell proliferation (Figure 1(a)). Colony formation indicated that overexpression of UCA1 promoted colony formation, while suppression of UCA1 inhibited colony formation of HL60 and U973 cells compared with the control group (Figure 1(b)). Transwell assays indicated that overexpression of UCA1 could promote invasion and migration while suppression of UCA1 inhibited HL60 and U973 cell invasion and migration (Figures 1(c) and 1(d)). Flow cytometry analysis showed that the apoptosis rates of HL60 and U973 cells were significantly decreased in the over-UCA1 group while increased in the Si-UCA1 group compared with the control group (Figures 2(a) and 2(b)). These results demonstrated that UCA1 promoted AML development *in vitro*.

### 3.2. METTL14 Influenced m6A Methylation Affected by lncRNA UCA1

**Figure 3:**
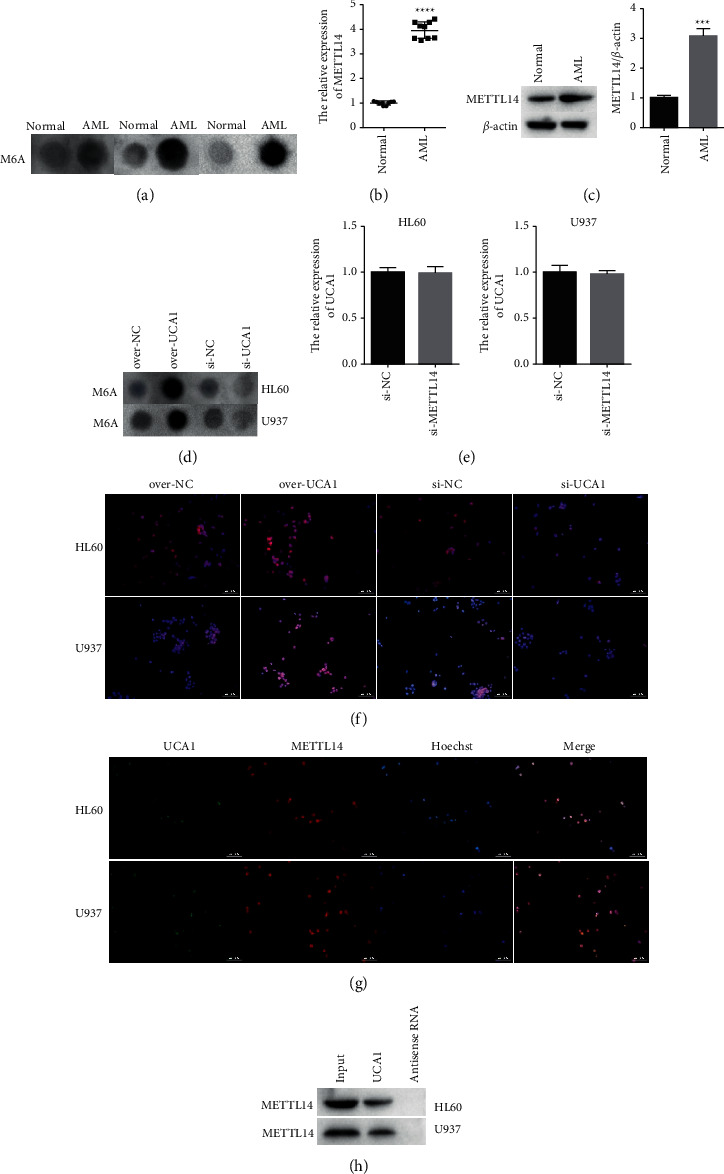
METTL14 influenced m6A methylation affected by lncRNA UCA1. (a) Dot blot assay was used to detect m6A methylation level in AML patients. (b, c) The expression of METTL14 in AML patients was detected by qRT-PCR and western blot, respectively. *β*-actin act as control. (d) m6A methylation level affected by UCA1 was detected by dot blot assay. (e) The expression of lncRNA UCA1 affected by suppression of METTL14 was detected by qRT-PCR. (f) Immunofluorescence staining was used to detect the expression of METTL14 affected by UCA1. (g) Colocalization of UCA1 and METTL14 in cytoplasm and nucleus. (h) RNA pull-down followed by western blot was used to detect the binding relationship between UCA1 and METTL14 protein. The data were presented by mean ± SD. ^*∗∗∗*^*P* < 0.005 and ^*∗∗∗∗*^*P* < 0.001.

m6A methylation plays important role in AML development [17]. We also demonstrated that m6A methylation level was significantly elevated in AML patients compared with normal controls (Figure 3(a)). Further analysis indicated that the expression of METTL14 was significantly elevated in AML patients compared with normal controls, as detected by qRT-PCR and western blot (Figures 3(b) and 3(c)). UCA1 overexpression increased the m6A level, while UCA1 knockdown reduced the m6A level (Figure 3(d)). Expression of UCA1 was not affected by suppression of METTL14 (Figure 3(e)), suggesting that METTL14 might be regulated by UCA1. Immunofluorescence staining indicated that the expression of METTL14 was dramatically increased in the over-UCA1 group while decreased in the Si-UCA1 group compared with the control group (Figure 3(f)). Further analysis indicated that UCA1 and METTL14 co-localized in both the cytoplasm and nucleus (Figure 3(g)). RNA pull-down results confirmed that UCA1 can bind with METTL14 protein (Figure 3(h)).

### 3.3. LncRNA UCA1 Affected m6A Methylation in AML Development

**Figure 4:**
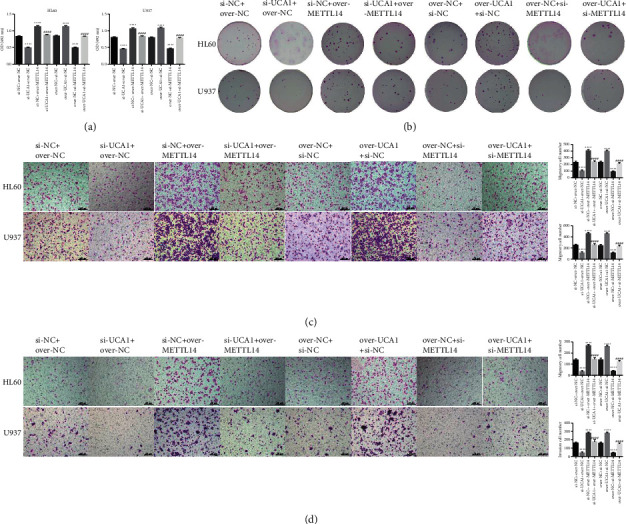
METTL14 rescued the effects of UCA1 on AML cell proliferation, invasion, and migration: (a–d) rescue experiment was used to detect the function of METTL14 on UCA1 on HL60 and U973 cell proliferation, colony formation, invasion, and migration, respectively. The data were presented by mean ± SD. ^*∗∗∗∗*^*P* < 0.001 and ^####^*P* < 0.001.

**Figure 5:**
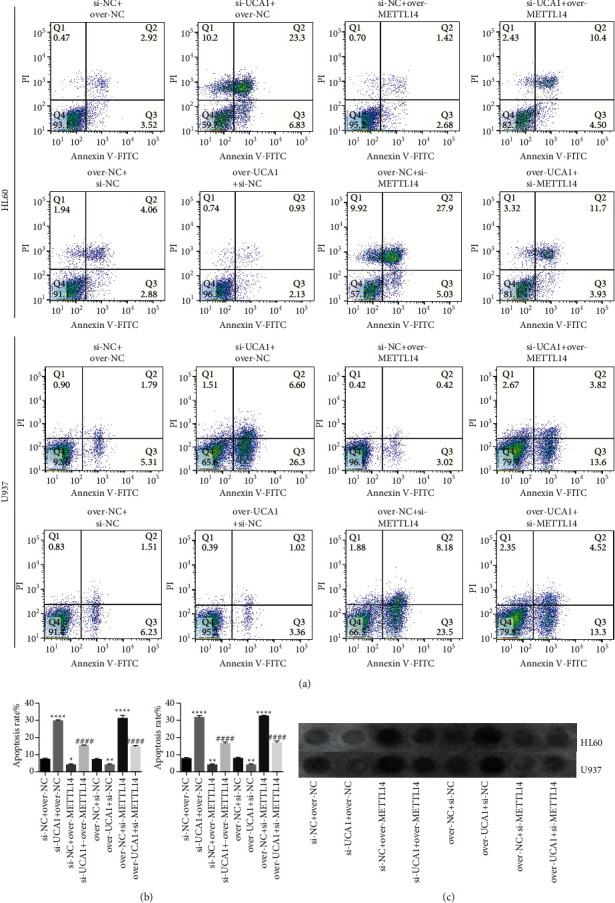
METTL14 rescued the effects of UCA1 on AML cell apoptosis and m6A methylation: (a, b) flow cytometry analysis was used to reveal the rescue effects of METTL14 on UCA1 on the apoptosis of HL60 and U973 cells and (c) dot blot analysis was conducted to show the rescue effects of METTL14 on UCA1 in m6A methylation. The data were presented by mean ± SD. ^*∗∗∗∗*^*P* < 0.001 and ^####^*P* < 0.001.

We also detected the function of METTL14 in UCA1-mediated AML development. The results indicated that UCA1-induced increase in HL60 and U973 cell proliferation, colony formation, invasion, and migration could be reversed by METTL14 (Figures 4(a)–4(d)). Flow cytometry analysis revealed that a decrease in HL60 and U973 cell apoptosis caused by lncRNA UCA1 overexpression or increase caused by lncRNA UCA1 suppression could be reversed by suppressing METTL14 or overexpressing METTL14 (Figures 5(a) and 5(b)). In addition, an increase in the m6A methylation level of HL60 and U973 cells caused by lncRNA UCA1 overexpression or a decrease caused by lncRNA UCA1 suppression could be reversed by suppressing METTL14 or overexpressing METTL14 (Figure 5(c)). These results demonstrated that lncRNA UCA1 affected m6A methylation participated in AML development.

### 3.4. The Expression of CYP1B1 and CXCR4 Was Affected by UCA1 and METTL14

**Figure 6:**
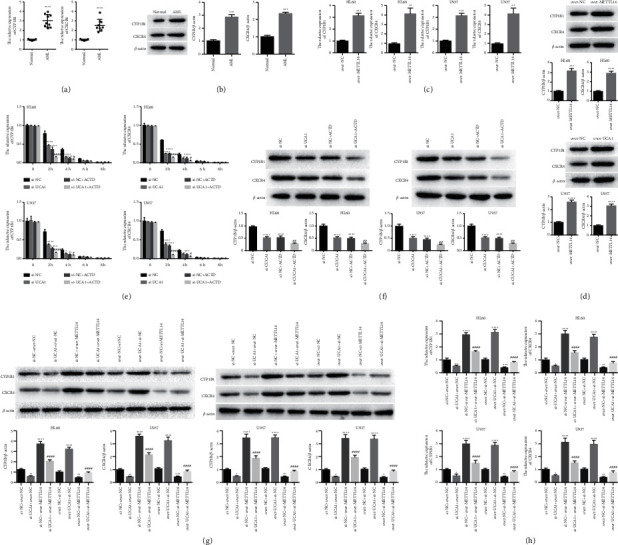
The expression of CYP1B1 and CXCR4 was affected by METTL14: (a, b) qRT-PCR and western blot were used to detect the expression of CYP1B1 and CXCR4 in AML patients; (c, d) the expression of CYP1B1 and CXCR4 affected by overexpressing METTL14 in both HL60 and U937 cells was detected by qRT-PCR and western blot; (e, f) the expression of CYP1B1 and CXCR4 affected by UCA1 and ACTD was assessed by qRT-PCR and western blot; and (g, h) the expression of CYP1B1 and CXCR4 affected by UCA1 and reversed by METTL14 was detected by western blot and qRT-PCR. The data were presented by mean ± SD. ^*∗*^*P* < 0.05, ^*∗∗*^*P* < 0.01, ^*∗∗∗*^*P* < 0.005, ^*∗∗∗∗*^*P* < 0.001, ^#^*P* < 0.05, ^##^*P* < 0.01, ^###^*P* < 0.005, and ^####^*P* < 0.001.

It has been proved that CYP1B1 and CXCR4 play important roles in AML [18, 19]. In order to know whether CYP1B1 and CXCR4 were affected by METTL14, the expression of CYP1B1 and CXCR4 was detected. qRT-PCR and western blot analysis indicated that the expression of CYP1B1 and CXCR4 was significantly elevated in AML patients compared with normal controls (Figures 6(a) and 6(b)). Furthermore, the expression of CYP1B1 and CXCR4 was greatly increased in the over-METTL14 group compared with the NC group in both HL60 and U937 cells (Figures 6(c) and 6(d)). Moreover, we found that suppression of UCA1 could decrease the expression of CYP1B1 and CXCR4, and the expression of CYP1B1 and CXCR4 was further decreased by treatment with ACTD for 2 and 4 h (Figure 6(e)), suggesting that suppression of UCA1 reduced the half-life of CYP1B1 and CXCR4. Western blot had also confirmed the results (Figure 6(f)). In addition, we demonstrated that the expression of CYP1B1 and CXCR4 was greatly increased in the over-UCA1 group while decreased in the Si-UCA1 group compared with the NC group. However, the expression changes of CYP1B1 and CXCR4 caused by UCA1 could be partially reversed by suppression of METTL14 or overexpression of METTL14 (Figures 6(g) and 6(h)). These results demonstrated that the expression of CYP1B1 and CXCR4 was affected by UCA1 and METTL14.

### 3.5. UCA1 Promoted AML Development by Affecting m6A Methylation *In Vivo*

**Figure 7:**
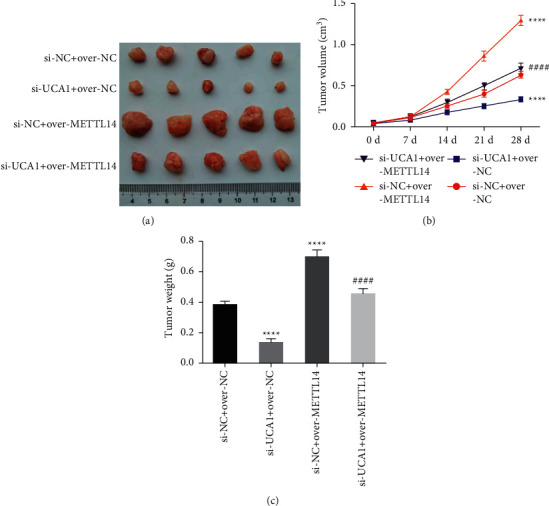
UCA1 promoted AML development by METTL14 *in vivo*: (a–c) effects of suppression of UCA1 and overexpression of METTL14 on tumor volume and tumor weight. The data were presented by mean ± SD. ^*∗∗∗∗*^*P* < 0.001 and ^####^*P* < 0.001.

**Figure 8:**
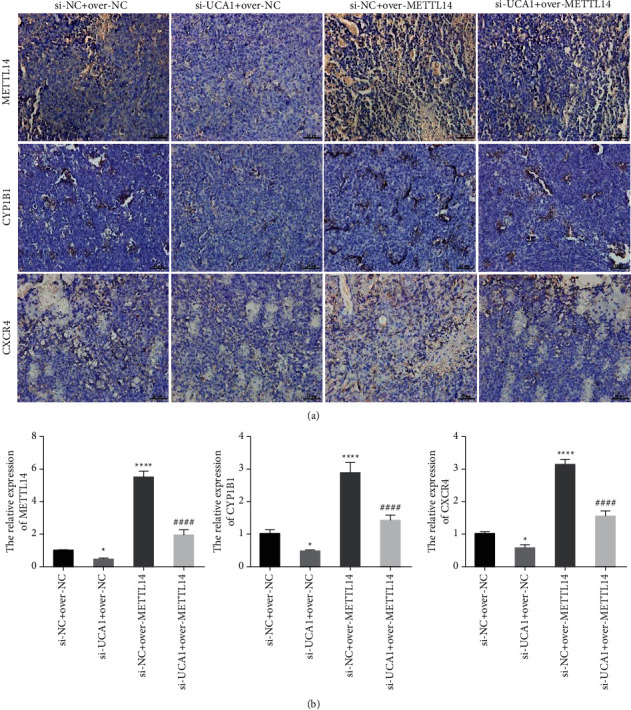
UCA1 promoted METTL14, CYP1B1, and CXCR4 expression *in vivo*: (a, b) the expression of METTL14, CYP1B1, and CXCR4 affected by suppression of UCA1, and overexpression of METTL14 was detected by immunohistochemical staining and qRT-PCR. The data were presented by mean ± SD. ^*∗*^*P* < 0.05, ^*∗∗∗∗*^*P* < 0.001, and ^####^*P* < 0.001.

We also detect the function of lncRNA UCA1 in AML development *in vivo*. Tumor volume and tumor weight were significantly smaller in the Si-UCA1 group while were dramatically increased in the over-METTL14 group compared with the control group. However, the smaller tumor volume and tumor weight caused by suppression of UCA1 could be partially reversed by overexpression of METTL14 (Figures 7(a)–7(c)). Furthermore, IHC and PCR analysis indicated that the expression of METTL14, CYP1B1, and CXCR4 was significantly downregulated in the Si-UCA1 group while was dramatically increased in the over-METTL14 group compared with the control group. However, the decrease in METTL14, CYP1B1, and CXCR4 expression caused by suppression of UCA1 could be partially reversed by overexpression of METTL14 (Figures 8(a) and 8(b)). These results demonstrated that lncRNA UCA1 promoted AML development by affecting m6A methylation *in vivo*.

## 4. Discussion

In the previous study, we demonstrated that lncRNA UCA1 modulated cell proliferation and apoptosis in AML [9]. However, the mechanism of UCA1 in AML development remains unclear. Several studies have proved that m6A methylation plays crucial roles in AML. In the present study, we revealed that UCA1 promotes the progression of AML by upregulating the expression of CXCR4 and CYP1B1 by affecting the stability of METTL14. This report innovatively revealed that m6A methylation is involved in AML development and is regulated by UCA1.

LncRNA is a transcript that is greater than 200 nucleotides in length and basically does not encode protein [20]. LncRNA plays a key regulatory role in almost all life activities such as cell proliferation and differentiation, ontogeny, signal transduction, stem cell maintenance, and metabolism, which contribute to various kinds of diseases [21, 22]. Liang et al. revealed that silencing of lncRNA UCA1 suppresses proliferation and accelerates apoptosis by repressing SIRT1 signals by targeting miR-204 in pediatric AML [23]. In consistent with the previous study, we demonstrated that overexpression of lncRNA UCA1 promotes AML development both in *vitro* and *in vivo*. Furthermore, UCA1 was found to positively regulate METTL14 expression by binding with METTL14 protein. Wen et al. demonstrated that METTL14 promotes leukemogenesis via m6A modification on its mRNA targets [24]. We found that METTL14 rescued the effects of UCA1 on AML development *in vitro* and *in vivo*, indicating that UCA1 regulates AML by positive modulation on METTL14.

Increasing studies have indicated that lncRNAs regulate disease initiation at the epigenetic level, transcription level, and post-transcription level. m6A methylation modification is one of the most common apparent modification methods of eukaryotic RNA [25]. Huang et al. revealed that FTO is a druggable target, and targeting FTO by small-molecule inhibitors holds the potential to treat AML [26]. Recently, Naren et al. demonstrated that high Wilms' tumor 1 associating protein expression predicts poor prognosis in AML and regulates m6A methylation of MYC mRNA [27]. In the present study, we found that the expression of METTL14 and m6A methylation levels were significantly elevated in AML patients compared with normal controls, suggesting that m6A methylation played a crucial role in the development of AML. Further analysis indicated that UCA1 positively regulated the m6A level. METTL14 is required for UCA1-mediated m6A levels. These results implied that UCA1 upregulates METTL14 to increase m6A levels and thus promotes AML.

It has been demonstrated that CXCR4 and CYP1B1 play crucial roles in AML and showed prognostic significance in AML [18, 28, 29]. Yang et al. indicated that knockdown of FTO increases m6A methylation in the critical protumorigenic melanoma cell-intrinsic genes including PD-1, CXCR4, and SOX10, leading to increased RNA decay through the m6A reader YTHDF2 [30]. Recently, Sun et al. demonstrated that LNC942 elicits potent oncogenic effects on promoting cell proliferation and colony formation and inhibiting cell apoptosis, subsequently elevating METTL14-mediated m6A methylation levels and its associated mRNA stability and protein expression of CXCR4 and CYP1B1 in breast cancer cells [31]. Compared with this study, we found that UCA1 can stabilize CXCR4 and CYP1B1 by binding with METTL14 protein in AML cells, which indicated the vital role of the METTL14/CXCR4/CYP1B1 pathway in AML. However, whether dysregulation of CXCR4 and CYP1B1 is the cause or the result of m6A methylation remains unclear and needs to be investigated. The METTL14-mediated m6Amethylation modification on which specific molecules deserved further exploration. Moreover, the stringency of our findings is limited by the small sample size.

## 5. Conclusion

In summary, we demonstrated that lncRNA UCA1 promoted the progression of AML by binding with METTL14 to increase m6A levels and upregulate the expression of CXCR4 and CYP1B1. These findings not only supplement the mechanism of lncRNA in AML but also provide new ideas for the treatment of AML.

## Data Availability

The data sets generated during and/or analyzed during the current study are available from the corresponding author on reasonable request.

## Supplementary Materials

Supplementary Materials
*Supplement Table 1*. Primers used in the present study.
