# Correction to “LncRNA UCA1 Promotes the Progression of AML by Upregulating the Expression of CXCR4 and CYP1B1 by Affecting the Stability of METTL14”

**DOI:** 10.1155/jo/9842168

**Published:** 2025-10-29

J. Li, Z. Li, X. Bai, et al., “LncRNA UCA1 Promotes the Progression of AML by Upregulating the Expression of CXCR4 and CYP1B1 by Affecting the Stability of METTL14,” *Journal of Oncology* 2022 (2022): 2756986, https://doi.org/10.1155/2022/2756986.

In the article, there are errors in Figure 1 which were introduced during the production process. Specifically:

• In Figure 1(c), the first graph was duplicated in the third graph.
• In Figure 1(c), the second graph was duplicated in the fourth graph.
• In Figure 1(f), the second graph was duplicated in the fourth graph.
• The second graph shown in Figure 1(g) represents HL60, not UL937.

Please find the correct Figure 1 below:

We apologize for these errors.

## Figures and Tables

**Figure 1:**
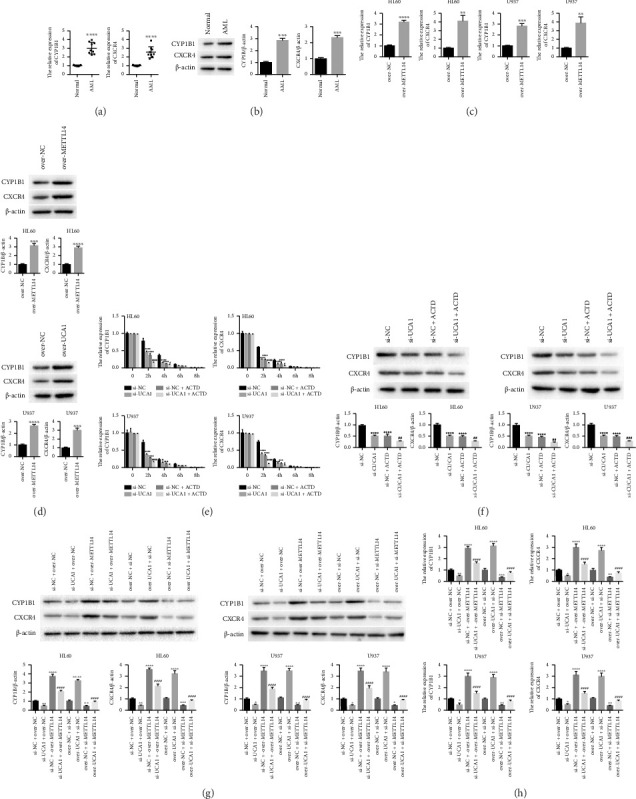
The expression of CYP1B1 and CXCR4 was affected by METTL14: (a, b) qRT-PCR and western blot were used to detect the expression of CYP1B1 and CXCR4 in AML patients; (c, d) the expression of CYP1B1 and CXCR4 affected by overexpressing METTL14 in both HL60 and U937 cells was detected by qRT-PCR and western blot; (e, f) the expression of CYP1B1 and CXCR4 affected by UCA1 and ACTD was assessed by qRT-PCR and western blot; and (g, h) the expression of CYP1B1 and CXCR4 affected by UCA1 and reversed by METTL14 was detected by western blot and qRT-PCR. The data were presented by mean ± SD. ^∗^*P* < 0.05, ^∗∗^*P* < 0.01, ^∗∗∗^*P* < 0.005, ^∗∗∗∗^*P* < 0.001, ^#^*P* < 0.05, ^##^*P* < 0.01, ^###^*P* < 0.005, and ^####^*P* < 0.001.

