# Supplementary material for: LncRNA UCA1 Promotes the Progression of AML by Upregulating the Expression of CXCR4 and CYP1B1 by Affecting the Stability of METTL14
Source: J Oncol. 2022 Feb 8;2022:2756986. doi: 10.1155/2022/2756986 (PMC8847036; doi:10.1155/2022/2756986)
Supplement: Supplementary Materials — Supplement Table 1. Primers used in the present study. [file 2756986.f1.docx]

**Supplement Table 1**

**Primers used in the present study**

| **Primers** | **Sequences (5' -3')** |
| --- | --- |
| METTL14-human-F | GAGTGTGTTTACGAAAATGGGGT |
| METTL14-human-R | CCGTCTGTGCTACGCTTCA |
| actin-human-F | AGCGAGCATCCCCCAAAGTT |
| actin-human-R | GGGCACGAAGGCTCATCATT |
| CXCR4-human-F | ACTACACCGAGGAAATGGGCT |
| CXCR4-human-R | CCCACAATGCCAGTTAAGAAGA |
| CYP1B1-human-F | AAGTTCTTGAGGCACTGCGAA |
| CYP1B1-human-R | GGCCGGTACGTTCTCCAAAT |
| UCA1-human-F | CTCAGAAGCCCCTTGGAC |
| UCA1-human-R | AGATGGACGGCAGTTGGT |
